# A Comprehensive Digital Program for Smoking Cessation: Assessing Feasibility in a Single-Group Cohort Study

**DOI:** 10.2196/11708

**Published:** 2018-12-18

**Authors:** Heather Patrick, Craig A Fujii, Debra B Glaser, David S Utley, Jennifer D Marler

**Affiliations:** 1 Carrot Inc Redwood City, CA United States

**Keywords:** smoking cessation, cell phone, health promotion, carbon monoxide

## Abstract

**Background:**

Cigarette smoking remains the leading cause of preventable death and disease worldwide. Evidence-based approaches are available, but few people access them. Although digital solutions offer great promise for population reach, few multicomponent programs exist. Pivot is a comprehensive digital solution combining a Food and Drug Administration–cleared carbon monoxide (CO) breath sensor; cigarette logging; a 6-phase, app-delivered smoking cessation program based on the US Clinical Practice Guidelines; and dedicated human coaching via text-based chat.

**Objective:**

The purpose of this study was to assess program engagement, changes in attitudes toward smoking, self-reported changes in smoking behavior, and program acceptability for the initial phase of Pivot: Explore.

**Methods:**

A total of 48 participants enrolled, and 41 completed the study. About half the participants (54%, 22/41) were men, and the mean age was 43 years. Most (85%, 35/41) were daily smokers and smoked an average of 12 cigarettes per day. Explore includes CO breath sensing, logging cigarettes in-app, learning via in-app activities, and dedicated human coaching through a text messaging interface. Participants completed surveys at baseline and exit assessing attitudes toward quitting including readiness, perceived difficulty, and confidence in quit success. At exit, participants also completed a survey of changes in smoking behavior and ratings of program acceptability.

**Results:**

More than 80% of participants (34-39 of 41) took ≥1 CO breath sample each day, and more than 55% (23-27 of 41) took ≥5 samples each day. More than 65% of participants (27-34 of 41) logged ≥1 cigarette using the in-app logging feature each day. All 9 in-app activities had completion rates ≥80% (33-40 of 41). Response to coach-initiated outreach was also high, with all contacts receiving ≥73% (30-39 of 41) response. In matched pair analyses, significant positive changes in mean attitudes toward quitting (scale 1-10) were evident from baseline (T1) to study exit (T2), including increased readiness to quit (T1 mean=6.1, T2 mean=7.4, *P*=.005), lower perceived difficulty (T1 mean=3.7, T2 mean=5.6, *P*=.001), and greater expectations of success (T1 mean=4.5, T2 mean=6.5, *P*<.001). At exit, 78% (32/41) of participants reported decreasing the number of cigarettes smoked per day during the study. Participants rated program quality and satisfaction very high (mean ≥8 for all items).

**Conclusions:**

These results support the feasibility and acceptability of the initial 9-day phase of Pivot: Explore. Participants had high levels of engagement with sensing, logging, learning, and coaching. Attitudes toward quitting improved significantly, and the majority of users indicated decreasing smoking behavior. Explore was designed to raise smoker awareness and motivation. Additional research is underway to assess how users progress through the full Pivot smoking cessation program and determine the program’s effectiveness for achieving sustained cessation.

## Introduction

### Background

Although cigarette smoking has declined considerably over the past 50 years, it remains the leading cause of preventable death and disease in the United States and worldwide. In the United States, approximately 36.5 million adults smoke cigarettes, and smoking-related illnesses are responsible for nearly 500,000 premature deaths annually [1]. Most people who smoke (70%) say they want to quit, and nearly half of them will make at least one quit attempt in any given year [2,3]. However, most smokers who try to quit (65%-75%) do so on their own and do not receive the support and assistance that would raise their chances of success. As a result, these unsupported quit attempts yield very low success rates (2%-5%) [4,5].

Traditionally, smoking cessation programs have been offered in-person or telephonically. In-person services are challenged by limited scalability, and appointment scheduling and transportation may create insurmountable participant burden, thus limiting reach [6-8]. Telephonic programs (ie, quitlines) are cost-effective and scalable, and they have been shown to increase the odds of successful smoking cessation [5,9,10]. However, these services have been underutilized [11].

Digital health interventions may help to alleviate these issues of reach, scalability, and access, particularly given the ubiquity of mobile phones across diverse populations. As of February 2018, 77% of US adults own and use a mobile phone, and 20% use their mobile phone as their primary means of accessing the internet. Mobile phone ownership is consistent across racial and ethnic groups, with 75% of blacks and 77% of both Hispanics and whites owning mobile phones. Dependency on mobile phones for internet access is higher among racial and ethnic minorities (35% Hispanics and 24% blacks vs 17% whites). Among lower-income populations (ie, those making less than US $30,000 per year), 67% own and use a mobile phone, and 31% of these individuals rely on their mobile phone for internet access [12].

In addition to their potential to improve reach, an emerging evidence base supports the efficacy of digital solutions for smoking cessation. A recent Cochrane review examined the literature including randomized or quasi-randomized trials using any type of mobile phone–based intervention for smoking cessation. A total of 12 studies were identified that included 6-month smoking cessation outcomes. Interventions were primarily text messaging based, although several included initial assessments or in-person visits along with text messaging. Results indicated that the evidence supports the positive impact of mobile phone–based smoking cessation interventions, most notably text messaging programs, on 6-month cessation outcomes [13]. Haskins et al conducted a systematic review to examine both the strength of the scientific evidence for smoking cessation mobile apps and the extent to which scientifically supported apps have been made commercially available [14]. Their review identified 6 apps with some level of scientific support, ranging from high quality (43%; exploratory pilot randomized controlled trials) to low quality (57%; acceptability or usability studies, feature-level analysis, or being grounded in an evidence-based approach but not subjected to a study). In the examination of commercially available smoking cessation apps, Haskins et al identified 177 unique apps relevant to smoking cessation in the App Store for iPhone and 139 in Google Play for Android. Only 3 of the 6 scientifically vetted apps were available in these app stores. Of these 3, only 2 were listed among the top apps by at least one app store.

Traditional smoking cessation programs delivered in-person or telephonically have demonstrated efficacy but limited reach and utilization. Digital solutions have greater potential for reach, and there is some evidence that they are efficacious, but few scientifically vetted apps have been designed for commercialization. Thus, their potential for reach may not be fully realized. In addition, for both traditional and digital smoking cessation programs, one of the early activities (often the first) involves setting a quit date and working on developing a quit plan. Although these are critical, evidence-based elements that all smoking cessation programs should include, requiring participants to select a quit date and actively work toward quitting from program entry may limit reach only to those who are ready to quit and have a reasonable degree of confidence in their ability to do so. There is an opportunity to develop and deliver interventions that include some *runway* before actively working toward a quit attempt, during which users are able to engage in self-exploration (eg, through self-monitoring) and reflect on how smoking fits into the bigger picture of their life. Programs that allow users to ease into quitting rather than starting with quitting may have a greater potential to reach the population of smokers who do not engage in other cessation programs and put them on the path to quitting.

Finally, to date, most digital solutions for smoking cessation have leveraged only 1 form of technology—typically text messaging or an app—which fails to capitalize on the breadth of technologies available to engage, motivate, and ultimately improve cessation rates among the population of people who continue to smoke. One exception to this has been carbon monoxide (CO) breath sampling. Several published studies [15-18], as well as expert opinion [19,20], suggest that digital sensors that provide individuals with their CO breath sample values can be educational and motivational and may lead to attitude changes—including increased interest in seeking a quit program. CO sampling is most commonly done in health clinics, using equipment that is not conducive to daily, real-time usage. A small, 10-participant study in the United Kingdom permitted smokers to self-administer breath samples to measure CO [15], but it was not integrated into an overall, evidence-based smoking cessation program.

### The Pivot Program

Pivot is a commercial-grade program designed for delivery in the context of employee wellness programs and health plans. It represents a comprehensive digital solution that brings together (1) the first Food and Drug Administration (FDA)–cleared (with over-the-counter labeling) CO breath sensor, which communicates via Bluetooth with a mobile phone and app; (2) a 6-phase mobile app delivering the US Clinical Practice Guidelines for Treating Tobacco Use and Dependence [5] and developed in collaboration with a team of scientific advisors representing some of the world leaders in tobacco control and smoking cessation; and (3) dedicated human coaching, delivered one-on-one through a digital text messaging interface.

Pivot is a year-long program designed to support users along the spectrum of quitting, from being unsure or on the fence about quitting to maintaining a smoke-free life. Pivot begins with *Explore*, which is designed for anyone who smokes, to raise awareness and interest in moving forward. In Explore, users take samples with the Pivot Breath Sensor, log cigarettes, get to know their coach, and complete daily activities to understand their smoking patterns and explore how smoking affects their lives. The second phase of Pivot is *Build*, which is tailored to users’ readiness, motivation, and confidence. Build culminates with users setting a quit date and building a quit plan. Next is *Mobilize*, which provides opportunities for users to put into practice individual elements of their quit plan, one at a time, in preparation for quit day. The fourth phase of Pivot is *Quit*, which begins on the user’s selected quit day and continues through the first week of living smoke-free. Quit incorporates a daily check-in feature to allow users to track their progress and set daily goals to reinforce the idea of quitting as a process. *Secure* is a natural extension of Quit and focuses on supporting users in developing internal, sustainable motivation to stay smoke-free for good. With continued coaching support, self-monitoring, and practice, Pivot’s newly smoke-free users learn to navigate the challenges that come in the first few months after quitting. The final phase of Pivot—*Sustain* —focuses on maintenance. Users continue to build skills and confidence and receive personal coaching designed to prevent relapse, so that they can remain smoke-free.

### Study Overview

The purpose of this study was to examine feasibility and acceptability of the initial, 9-day phase of the Pivot program—Explore. One of the defining elements of Explore is that unlike most cessation interventions (digital or otherwise), the program does not begin with setting a quit date and building a quit plan. Instead, Explore is designed for all smokers, whether they are ready to quit or not. To that end, Explore consists of 4 key components: (1) use of a CO breath sensor; (2) in-app cigarette logging; (3) activities that encourage self-exploration; and (4) interaction with a dedicated human coach through a text messaging interface. These components are well grounded in the literature, and Pivot expands on existing digital interventions by bringing these components together in a single, integrated solution. As noted above, CO breath sampling has been shown to be educational and motivational and may be particularly useful early on in cessation programs and particularly for those individuals who are not yet ready to quit smoking [19,20]. Unlike sensors that have traditionally been used in clinical settings, Pivot’s portable, Bluetooth-enabled breath sensor allows for daily sampling and feedback. Self-monitoring via cigarette logging is a commonly used evidence-based behavior change strategy implemented in many cessation programs [5]. In Explore, participants can easily log cigarettes in the Pivot app and see visual displays of their smoking patterns, including the amount and times of day when they are most likely to smoke. The activities in Explore leverage principles of motivational interviewing [21] to help participants move from being unsure or ambivalent about quitting smoking to being ready to work toward quitting. For example, some activities allow participants to explore how smoking affects their lives in terms of time and financial costs, including creating an opportunity to connect to their broader values by considering how they might otherwise spend those resources if not on smoking. Other activities encourage participants to consider how smoking might serve a purpose in their lives, including identifying their reasons for smoking. Finally, in Explore, participants work with a dedicated human coach via a text messaging interface. Behavioral counseling is a pillar of evidence-based smoking cessation programs [5]. However, behavioral counseling is often underutilized because of challenges with scheduling, transportation, or both. In addition, many telephonic programs do not allow participants to interact with the same coach over time, thus limiting the potential for participants to establish a strong therapeutic relationship with their cessation counselor. In Explore—and throughout Pivot—coaching is designed to directly address these barriers by (1) mitigating the need for scheduling by providing asynchronous chat, which allows participants to respond to coach-initiated outreach at their convenience, (2) eliminating both transportation and scheduling requirements by allowing participants to message their coach anytime and anywhere, and (3) creating the opportunity to cultivate a strong coach-participant relationship by assigning participants a dedicated coach so they are working with the same person each time they connect with their Pivot coach.

## Methods

### Study Design

This was an open-label, single-group, pretest-posttest study of the initial, 9-day phase of the Pivot program—Explore. The study was conducted as a feasibility and acceptability study to examine levels of engagement with program elements (ie, sensing, logging, learning, and coaching), analyze changes in attitudes toward quitting smoking from baseline (T1) to study exit (T2), and describe self-reported changes in smoking behavior and satisfaction with Pivot.

### Recruitment and Study Population

Participants in the greater San Francisco Bay Area were recruited via Web-based advertisements and a clinical study recruiter. Participants completed a telephone screener to determine eligibility and receive a description of the study. Eligibility criteria included being aged 27 to 57 years, being able to speak and read English, smoking 5 or more cigarettes per day, owning and using a mobile phone (iPhone 5 and above, operating system iOS 9.0 and above, or Android 4.4 and above, operating system Android 4.4 and above), and using at least 1 app on their mobile phone. All participants indicated that they worked ≥30 hours per week. Thus, all participants were benefits-eligible (ie, eligible to receive insurance, wellness, and other benefits through their employer). Participants represented a range of employment sectors, including sales, warehouse management, human resources, forklift operation, guest services, administrative or secretarial, and education or teaching. According to 2015 data from the Society for Human Resource Management [22], approximately 70% of US employers offer some form of wellness benefit to employees—either through a stand-alone wellness program or through insurance benefits. These wellness benefits are offered across a range of employment sectors, including those represented by this participant population. Thus, this sample is representative of the Pivot target market. Participants did not have to indicate an intent to quit smoking as a condition of study participation. Interested, eligible participants were invited to attend an on-site study appointment.

**Figure 1:**
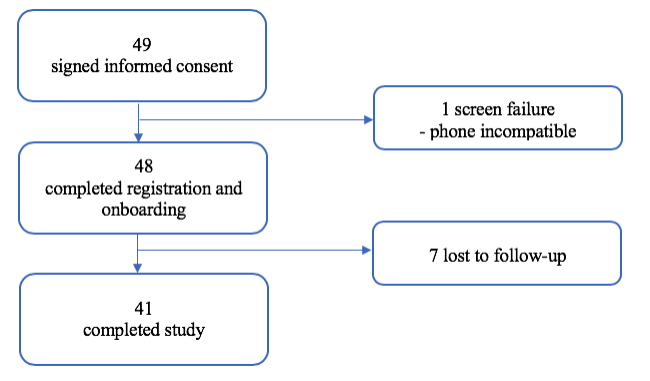
Consort (Consolidated standards of reporting trials) flow diagram of participants.

Figure 1 provides the CONSORT diagram for the study. As shown, 49 potential participants attended the study site visit and provided informed consent. One participant did not have a compatible phone; thus, 48 participants were enrolled, and 41 participants completed the study. Overall, 7 participants who were lost to follow-up were either not reachable after at least three contact attempts or did not agree to return. Moreover, 2 participants stated they smoked 5 or more cigarettes per day during the phone screening; however, they indicated that they smoked 4 cigarettes per day during registration. It is possible that these participants decreased smoking from screening to day 1.

As this was a feasibility and acceptability study, the purpose was to examine how people engaged with this first phase of the Pivot program, before developing the full Pivot experience, to inform the development of later phases of the program and to understand how and whether this first phase of the program was associated with shifts in attitude or behavior that might suggest the potential for participants to (1) engage in later phases of the Pivot program and (2) attempt to quit or quit smoking. As hypothesis testing was not a primary aim of this study, we did not conduct power analyses to determine sample size. Rather, we recruited and enrolled a sample size that allowed us to glean the necessary insights for further program development and additional research and ceased enrollment once we reached saturation. This is consistent with emerging standards for feasibility and pilot work [23].

### Consent and Ethical Approval

All participants provided written and oral informed consent before participation. The study protocol was reviewed and approved by Solutions Institutional Review Board (Little Rock, AR, USA), Protocol #2017/04/12.

### Procedure

During the on-site appointment, participants provided informed consent and then used a study laptop to complete the Web-based study registration and a baseline questionnaire to assess smoking history (ie, age when the participant started smoking, number of years smoked), number of cigarettes smoked per day, and attitudes toward quitting. They were then provided with the CO breath sensor and instructions to self-train on the use of the sensor and to download the Pivot app on their mobile phone. The CO breath sensor is FDA-cleared for single-user use by cigarette smokers in smoking cessation programs to inform the user about how breath CO levels are affected by smoking behavior. During the 9-day study period, participants were asked to engage with Explore, which comprised all features of the Pivot program: sensing, logging, learning, and coaching.

#### Sensing

**Figure 2:**
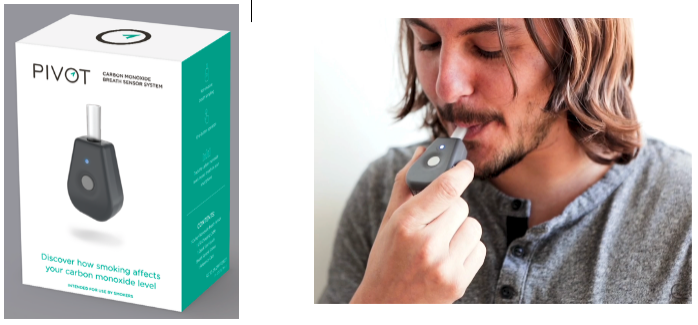
The Pivot carbon monoxide (CO) breath sensor.

Participants were instructed to use the CO breath sensor to complete hourly breath samples while awake. In the Pivot app, they were able to view their breath sample results, including the CO levels of each breath sample. Figure 2 shows the CO breath sensor and how it is used.

#### Logging

The Pivot app includes a cigarette-logging feature, and participants were asked to log all cigarettes smoked and were encouraged to log as soon after smoking as possible. The purpose of logging was to facilitate awareness of smoking behavior. The Pivot app provides participants with a visual display of smoking trends, including information about number, time, and location of cigarettes smoked.

#### Learning

**Figure 3:**
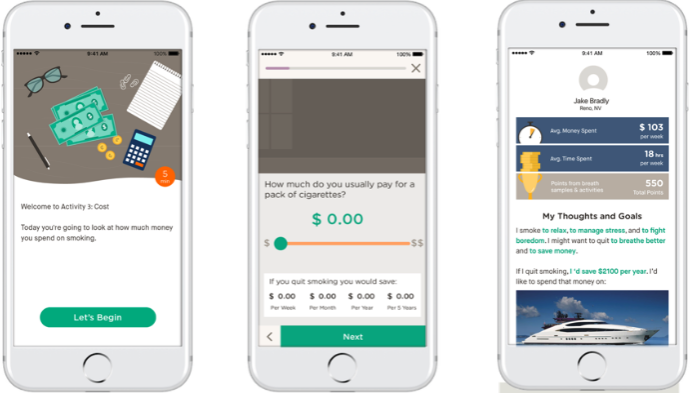
Example Pivot activity.

The Explore phase of Pivot consists of 9 activities. One activity was *unlocked* each day, and participants received a daily push notification letting them know when a new activity had become available. The first 2 activities in Explore focus on CO and provide an overview of what CO is, how it is related to smoking, and why measuring it with the CO breath sensor is important. The remaining activities provided additional opportunities for participants to learn about how smoking fits into and influences their daily lives. This included a cost calculator, identifying both reasons for smoking and reasons for considering quitting, assessing level of addiction, exploring one’s household influences, a calculation of time spent smoking, self-reflection on the importance of and confidence about quitting, and a summary of the participant’s experience and activity in Explore. Figure 3 provides an example of one of the activities from Explore.

#### Coaching

Participants interacted with their dedicated coach via an asynchronous, text messaging interface. Coaches initiated 4 contacts during Explore: 1 after participants completed onboarding, 1 on day 3 or 4, 1 on day 5 or 6, and the last on day 8 or 9, before participants exited the study. Coaching conversations focused on supporting participants in reflecting on their experience in Explore, including what they were learning from using the CO breath sensor, logging cigarettes, and completing activities.

On day 9, participants returned to the study site to complete the final activity in the Pivot app and the end-of-study survey. The end-of-study survey included measures of attitudes toward quitting, self-reported changes in smoking behavior, and satisfaction with Pivot. Participants received a US $100 stipend on completion of the study. Participants were provided the stipend to compensate them for time and transportation costs associated with attending 2 onsite study visits and for the time spent participating during the 9 days of the study. All participants received this stipend, regardless of the level of engagement with Pivot.

### Outcome Variables and Measurement

#### Engagement

Program engagement was assessed across all 4 elements of Explore: (1) sensing: use of the CO breath sensor (average daily breath samples and percentage of study population using the breath sensor); (2) logging: use of the in-app cigarette logging feature (average number of cigarettes logged in-app per day and percentage of study population using the logging feature); (3) learning: completion of daily in-app activities; and (4) coaching: response to coach-initiated outreach via texting interface.

#### Attitudes Toward Quitting Smoking

Participants answered 3 items to assess attitudes toward quitting smoking at baseline (T1) and study exit (T2), selecting a number using a 1 to 10 scale. Items included: *If you were to quit smoking right now, how difficult do you think it would be to stay smoke-free?* (1=really hard; 10=really easy); *If you were to quit smoking right now, how successful would you be?* (1=not successful at all; 10=completely successful); and *How ready are you to quit smoking?* (1=not ready at all; 10=completely ready).

#### Self-Reported Smoking Behavior

At study exit, participants answered several questions regarding whether and how their smoking behavior had changed over the course of the study. These items included the following: Do you feel that the amount you smoke has changed since your first study visit, 9 days ago? (Yes or No); (If Yes) Do you feel the amount decreased? (Yes or No); (If Yes) How did you decrease it? Select all that apply (I increased my time between cigarettes; I smoked less of each cigarette; I smoked fewer cigarettes per day; Other); (If Yes) What was the change due to? Read all options and select the single best answer (Being prompted to submit breath samples hourly [first 7 days of study]; Realizing how much money I spend on smoking; Seeing *the [CO] guy* fill up with red; Tracking my CO levels; Realizing the time I spend smoking; Tracking my cigarettes; Other).

#### Satisfaction With Pivot

At study exit, participants answered 3 items to indicate their satisfaction with Pivot. All items were answered by selecting a number from 0 to 10, as described below. Items included: *How informative did you find the program?* (0=not at all informative, 10=very informative); *How would you rank your satisfaction with the product?* (0=not at all satisfied, 10=very satisfied); and *How likely are you to recommend this program to a friend?* (0=not at all likely, 10=very likely). Likelihood of recommending Pivot to a friend was converted to a net promoter score (NPS). NPS is an industry indicator of participant loyalty to a product or service. NPS was calculated by subtracting the percentage of respondents who answered 6 or lower (detractors) from the percentage of respondents who answered 9 or 10 (promoters). Finally, participants indicated how many times per day they would be willing to use the breath sensor as part of a smoking cessation program.

### Statistical Analyses

Statistical analyses were conducted using data from the 41 participants who completed the study. For engagement, data were collected through the Pivot app to describe sensing (eg, average daily breath samples), in-app cigarette logging, and completion of in-app activities. Response to coach-initiated contact was measured via the text messaging interface to indicate whether a participant responded to a coach-initiated message sent as part of the 4-coach touchpoint protocol described above. Analyses were conducted to calculate the mean (SD) for normally distributed variables or median (interquartile range) values in instances of non-normally distributed variables. Analyses involving attitudes toward quitting smoking were conducted with matched-pair *t* tests using SAS Version 9.4 (SAS Institute, Cary, NC, USA) to determine whether statistically significant changes in attitudes occurred from baseline (T1) to study exit (T2). Statistical significance was set at *P*<.05. Self-reported change in smoking behavior was analyzed as the percentage of study participants who indicated that they changed their smoking from the beginning to the end of the study. Finally, satisfaction with Pivot was analyzed descriptively to reflect mean and SD of responses for items on degree of program informativeness and program satisfaction. NPS was calculated for the item reflecting likelihood of recommending Pivot to a friend.

## Results

Per-protocol analyses were conducted using the 41 participants who completed the study. More than half of study participants (54%, 22/41) were men, and the average age of participants was 43 years (SD 9 years). Most participants (61%, 25/41) used Android mobile phones, and most participants (85%, 35/41) smoked daily. Baseline characteristics of smoking history and experience are presented in Table 1.

**Table 1:** Baseline characteristics of smoking history and experience (n=41).

Characteristics	Mean (SD)
Age when started smoking	21.0 (10.2)
Years smoking	21.4 (13.0)
Cigarettes smoked per day	12.2 (6.0)

### Engagement

#### Sensing

**Figure 4:**
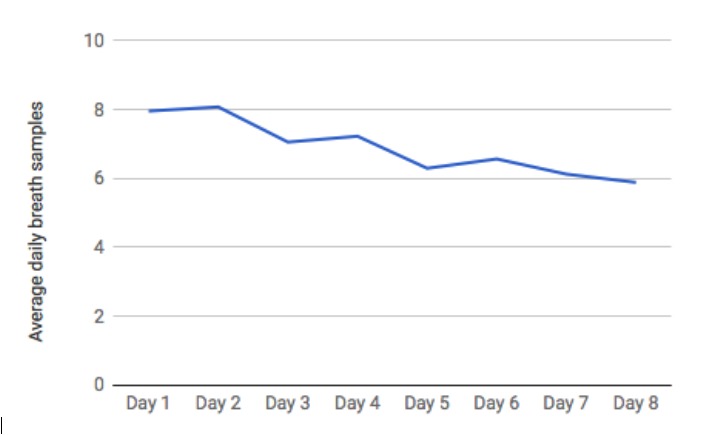
Average daily breath sensor usage.

Figure 4 presents average daily breath sensor use for all participants (n=41) across days 1 to 8 of the study. On day 9, participants returned to the study site and therefore did not have a full day available for sensor use. As shown, self-monitoring via the breath sensor was reasonably consistent throughout the study, ranging from a high of 8.1 breath samples on day 2 (the first full day of participation) to a low of 5.9 breath samples on day 8 (the last full day of participation). The interquartile range (25%-75%) was 2 to 12 samples per day.

**Figure 5:**
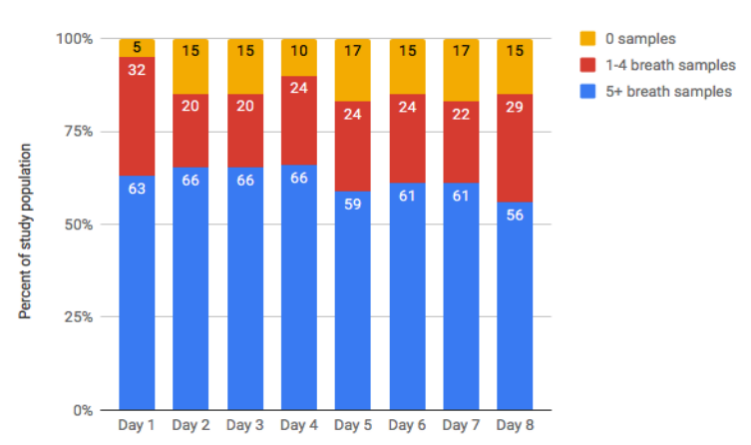
Percentage of participants using the breath sensor 0, 1 to 4, and more than 5 times each day.

Figure 5 shows, for each day of the study, the percent of the study population who used the breath sensor 0, 1 to 4, and 5 or more times on each day of the study. Overall, daily breath sensor use was quite high, with 83% to 95% of participants (34-39 of 41) using the breath sensor at least once daily.

#### Logging

**Figure 6:**
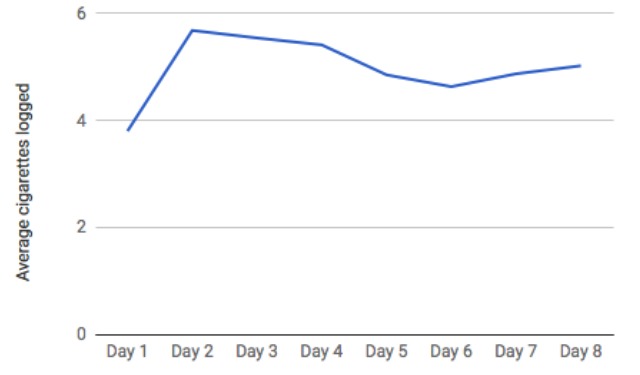
Average cigarettes logged in-app per person per day.

**Figure 7:**
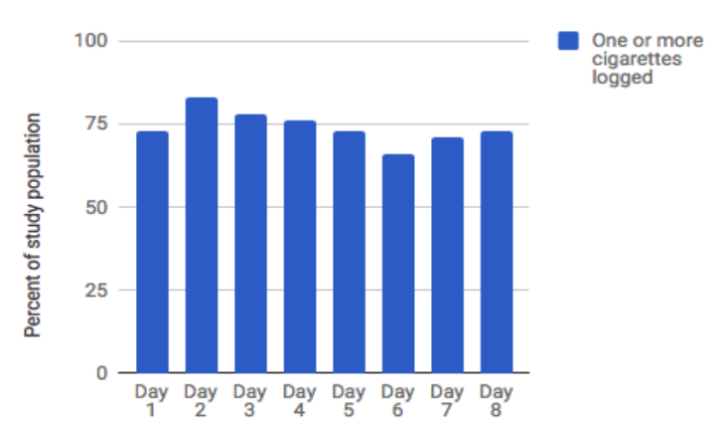
Percentage of study population logging at least one cigarette on each day.

Figure 6 presents the average number of cigarettes logged per day, using the in-app logging feature. Data include all participants (n=41) across days 1 to 8 of the study. As shown, beginning on day 2 (the first full day of study participation), use of the in-app logging feature was quite consistent, with participants logging 4.6 to 5.7 cigarettes per day, on average. The interquartile range (25%-75%) was 0 to 9 cigarettes logged per day. Figure 7 presents the percent of the study population who logged at least one cigarette each day using the in-app logging feature. For each day of the study, more than 65% of the study population (27-34 of 41) logged at least one cigarette using the in-app logging feature.

#### Learning: Completion of In-App Activities

**Figure 8:**
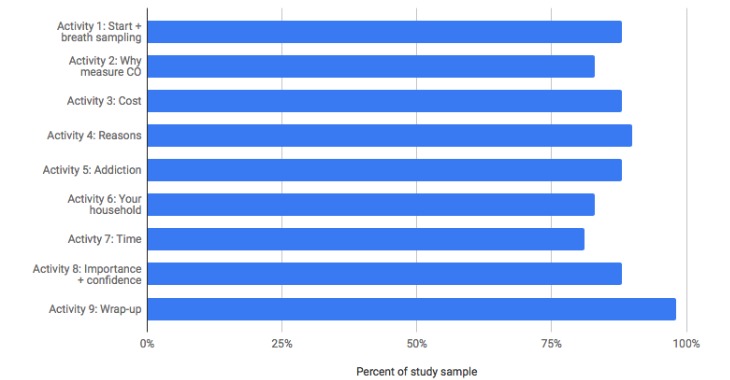
Percent completion of in-app activities. CO: carbon monoxide.

Figure 8 presents percent completion for each of the 9 daily, in-app activities. Participants who had not yet completed activity 9 when they attended the on-site study exit interview were asked to do so before exiting the study. Thus, this activity shows the highest completion rate. For all remaining activities, participants completed them on their own, within the context of their daily lives. As shown, completion rates were high, with each activity having a completion rate of 80% or higher (33-40 of 41).

#### Coaching Engagement

Table 2 presents data on engagement with coaching. As shown, each coach touchpoint had a participant response rate of 73% or greater (30-39 of 41). Coach-initiated touchpoints were conducted via a digital text messaging interface. The 2 columns on the right in Table 2 present mean number of messages per touchpoint from coaches and participants, respectively.

**Table 2:** Engagement with human coaching via digital text messaging interface.

Touchpoint	Participants responded, n (%)	Coach-sent messages, mean (SD)	Participant-sent messages, mean (SD)
1	39 (95)	9.8 (4.4)	6.7 (4.1)
2	31 (76)	8.6 (5.2)	6.0 (5.5)
3	32 (80)	7.8 (7.0)	4.7 (5.6)
4	30 (73)	6.7 (6.3)	3.9 (5.1)

### Change in Attitudes Toward Quitting

Table 3 presents results from matched-pair analyses on changes in attitudes toward quitting smoking from baseline (T1) to end of study (T2). As shown, there were significant, positive changes in attitudes toward quitting smoking such that at study exit, participants expected lower difficulty in quitting, had greater confidence in success, and were more ready to quit.

### Self-Reported Change in Smoking Behavior

More than three-fourths of the study population (78%, 32/41) indicated that their smoking behavior had changed from the beginning of the study to the end. Of participants who reported changing their smoking behavior, nearly all (31/32) indicated that they had decreased the amount that they smoked. The most common means by which participants decreased smoking included smoking fewer cigarettes per day (87%, 27/31) and increasing time between cigarettes (39%, 12/31). When asked what the change in smoking behavior was due to, the most common responses were tracking my cigarettes (45%, 14/31), tracking my CO levels (35%, 11/31), and being prompted to submit hourly breath samples (32%, 10/31).

### Satisfaction With Pivot

**Figure 9:**
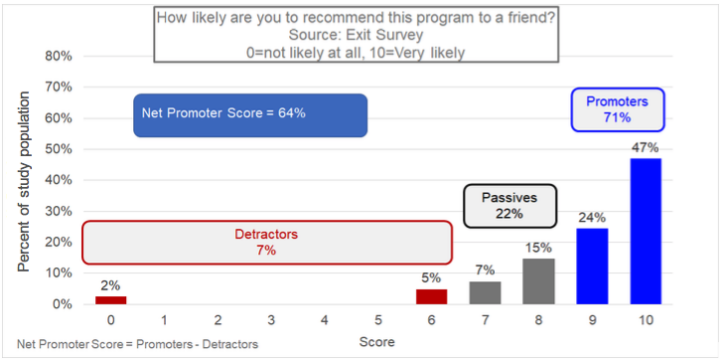
Net promoter score (NPS).

Overall, participants indicated that they thought the Pivot program was very informative (mean=8.4, SD=1.7) and that they were quite satisfied with the program overall (mean=8.5, SD=1.8). Figure 9 presents the distribution of responses to the question *How likely are you to recommend this program to a friend*, which was used to calculate NPS. As shown, the NPS score for the Explore phase of Pivot was 64. This is considered *excellent* using global NPS standards [24]. Most health apps do not report NPS publicly. As a point of reference from other health-relevant vendors, BlueStar (a product of Welldoc) reports an NPS of 70 [25], WebMD reports an NPS of 60 [26], and in a recent survey on NPS for primary care, patients indicated an NPS of 30 [27]. Finally, regarding willingness to use the breath sensor as part of a cessation program, on average, participants indicated they were willing to provide 10.3 breath samples per day. The interquartile range (25%-75%) was 6.5 to 12.0 samples per day.

**Table 3:** Changes in attitudes toward quitting smoking. For paired change analyses, positive numbers are favorable.

Attitude toward quitting	T1 (baseline), mean (SD)	T2 (end of study), mean (SD)	Paired change (SD)	*P* value
Difficulty	3.7 (3.2)	5.6 (2.7)	1.9 (3.4)	.001
Success	4.5 (2.7)	6.5 (2.6)	2.1 (3.0)	<.001
Readiness	6.1 (3.0)	7.4 (2.5)	1.2 (2.6)	.005

## Discussion

### Principal Findings

This was a feasibility study of the first phase of the Pivot program, Explore. Digital solutions offer one mechanism by which evidence-based approaches to smoking cessation can be disseminated at scale. Consistent with recommendations for features likely to increase program effectiveness, Pivot has been designed to offer the following: social context and support (coaching), multiple means of contact with the intervention (sensing, logging, learning, and coaching), tailoring (sensing and coaching), and self-management (sensing and logging) [28]. During the 9-day study, participants used all 4 program features: sensing via the personal mobile CO breath sensor, logging cigarettes, learning through completion of in-app activities, and coaching via a digital text messaging interface. They completed measures of attitudes toward quitting smoking at baseline and at the end of the study, and at the end of the study, answered questions about changes in smoking behavior and satisfaction with the program.

### Program Engagement: Sensing, Logging, Learning, and Coaching

Across all program components, engagement was quite high. More than 80% of participants used the CO breath sensor at least once on each day of the study. At least two-thirds of participants used the in-app cigarette logging feature daily. Each of the 9 in-app activities had completion rates of 80% or higher, and responsiveness to each of the 4 coach-initiated touchpoints was 73% or higher.

This feasibility study provides initial support that participants are willing and able to engage with all components of Pivot’s comprehensive evidence-based solution. This finding is encouraging, given the consistent dose-response relationship found in smoking cessation interventions, in which greater exposure to the intervention and in this case, multiple modes by which to have such exposure, is likely to improve cessation outcomes [5,28]. An important question to examine in future work is what constitutes *effective engagement* in Pivot (ie, the degree of engagement likely to yield the intended behavior change) [29]. Part of this will involve identifying the ideal amount and combination of engagement with each program component to yield clinical outcomes such as quit attempts and sustained cessation. It is also likely that different user profiles will emerge such that some components (or combinations of components) are particularly beneficial for different users based on user preferences, smoking history, readiness to change, and other characteristics. As we collect more data on user behavior and engagement with Explore and the remaining phases of Pivot, we will be able to examine different usage patterns. Different usage patterns may be related to baseline user characteristics such as smoking history and attitudes toward quitting, as well as subsequent quitting behavior including setting a quit date, building a quit plan, making quit attempts, and sustaining quits over time. This is a longer-term endeavor that will require much larger sample sizes and leveraging data collected surreptitiously through the app as well as user-provided data in the form of baseline and in-app surveys.

### Changes in Attitudes and Self-Reported Behavior

The study also demonstrated positive, statistically significant improvements in three indicators of attitudes toward quitting smoking: increased readiness to quit, increased anticipation of success, and reduced perceptions of difficulty quitting. These attitude shifts are meaningful, considering the role of motivational factors (motivation to quit, confidence in quitting) in predicting quit attempts [30]. Well over half of participants demonstrated improvements in readiness (58%, 23/41), anticipation of success (73%, 29/41), and reduced perceptions of difficulty (65%, 26/41). In previous research, only 15% to 34% of participants demonstrated short-term changes in attitudes toward quitting over time frames ranging from 8 to 30 days [31-34]. It is possible that participants adopted other strategies—such as the use of nicotine replacement therapy—to support changes in attitudes and behavior. However, nothing in the Explore phase of Pivot directly focuses on cigarette reduction, strategies for dealing with cravings or withdrawal, or the use of medications to support reducing or quitting smoking.

In addition, more than three-quarters of participants indicated that they had reduced the number of cigarettes they smoked during this 9-day experience. These findings are particularly promising, given that the focus of the first phase of Pivot is not intended to help users reduce or quit smoking. Rather, the purpose of the first 9 days of the program is to provide participants with an opportunity to learn about how smoking fits into their life and about how smoking and CO levels are linked. The fact that the experience of self-exploration and learning was associated with positive changes in how people feel about quitting smoking and with self-reported changes in smoking behavior suggests potential for the full Pivot program to lead to smoking cessation. In addition, because Pivot has been designed with this 9-day on-ramp that does not start with setting a quit date or working on a quit plan, Pivot may be uniquely positioned to appeal to smokers who otherwise would not participate in a cessation program.

### Program Satisfaction and Acceptability

Satisfaction with and acceptability of Pivot was very high across multiple indicators including program informativeness, satisfaction, and NPS. As a metric of user loyalty, NPS is particularly important because Pivot is likely to be more effective with achieving clinical outcomes (ie, quit attempts and sustained cessation) for people who progress through later phases of the program. The fact that users indicated a high degree of loyalty for the first phase of Pivot shows promise for the potential longer-term *stickiness* of the program [24].

### Limitations

This was a small feasibility study that examined only the first phase of a 6-phase smoking cessation program. Thus, we did not assess cessation outcomes. Participants were recruited from the San Francisco Bay Area, where policy, taxation, and social norms around smoking are more stringent than in other parts of the United States. In addition, given the influence of the technology sector in this area, participants were likely more tech-savvy than maybe expected elsewhere. Participants received a stipend (US $100) in exchange for their participation in the study. It is possible that this could have biased the findings. However, (1) the stipend was paid to them on completion of the final study assessment, regardless of their level of engagement in Pivot during the 9 days of the study, and (2) participants were told that the stipend was intended as a thank you for their time as a study participant and to cover transportation time and expenses for 2 trips to the study site. Thus, the stipend was not contingent on their levels of engagement with Pivot. Finally, although engagement across all components of the program was quite high, it is worth noting that utilization of the self-monitoring features (ie, sensing and logging) was somewhat lower than anticipated. With regard to the use of the CO breath sensor, participants were instructed to use the sensor hourly while awake; however, they used the sensor an average of 5.9 to 8.1 times per day. Use of the cigarette logging feature was also somewhat lower than anticipated. Participants reported smoking an average of 12.2 cigarettes per day at baseline and recorded 4.6 to 5.7 cigarettes daily using the in-app logging feature. Although possible, it seems unlikely that participants reduced smoking that drastically starting with the first full day of the study. Thus, we were unable to use cigarette logging as an indicator of smoking behavior and instead relied on participant’s self-reported changes in smoking. This is similar to what others have found in a mobile app that includes cigarette-logging features, which have been used to support participants in cultivating awareness of behavioral patterns of smoking, rather than as an objective measure of cigarettes smoked [35]. A question on cigarettes smoked per day or the validated Fagerstrom test would have offered a more robust indication of behavioral shift. Despite the somewhat-lower-than-anticipated engagement with the self-monitoring features of Pivot, it is worth noting that using the breath sensor and tracking cigarettes were two of the most commonly noted features that participants felt contributed to their self-reported changes in smoking behavior.

### Conclusions

Despite these limitations, the findings from this study provide support for the feasibility of the initial phase of Pivot: Explore. Pivot brings together a unique, comprehensive combination of technologies including the FDA–cleared CO breath sensor, evidence-based content presented through an engaging mobile app, and dedicated human coaching delivered via text messaging interface. Engagement across all program components was high as was program satisfaction and acceptability. In addition, the statistically significant, positive changes in attitudes toward smoking and self-reported changes in smoking behavior are promising, particularly because the first phase of Pivot does not directly address or promote smoking cessation. To confirm the promising results of these initial findings, additional research is underway to examine engagement with and progression through the full Pivot journey and evaluate program effectiveness for quit attempts and short- and long-term cessation outcomes. In addition, research is underway to better understand optimal program engagement and begin to identify and tailor on user profiles.

## Abbreviations

CO: carbon monoxide
FDA: Food and Drug Administration
NPS: net promoter score
